# Can Sierra Leone maintain the equitable delivery of their Free Health Care Initiative? The case for more contextualised interventions: results of a cross-sectional survey

**DOI:** 10.1186/s12913-016-1496-1

**Published:** 2016-07-13

**Authors:** Frédérique Vallières, Emma Louise Cassidy, Eilish McAuliffe, Brynne Gilmore, Allieu S. Bangura, Joseph Musa

**Affiliations:** Centre for Global Health, Trinity College Dublin, University of Dublin, 7-9 Leinster Street South, Dublin 2, Ireland; School of Psychology, Trinity College Dublin, University of Dublin, 2 College Green, Dublin 2, Ireland; School of Nursing, Midwifery and Health Systems, College of Health Sciences, University College Dublin, Belfield, Dublin 4, Ireland; World Vision Sierra Leone, 35 Wilkinson Road, Freetown, Sierra Leone

**Keywords:** Maternal and child health, Sierra Leone, Free health care, Bonthe District, Ebola

## Abstract

**Background:**

In 2010, the Ministry of Health and Sanitation in Sierra Leone launched their Free Health Care Initiative (FHCI) for pregnant and lactating mothers and children under-5. Despite an increase in the update of services, the inequitable distribution of health services and health facilities remain important factors underlying the poor performance of health systems to deliver effective services. This study identifies current gaps in service delivery across two rural locations served by the same District Health Management Team (DHMT).

**Methods:**

We employed a cross-sectional household survey using a two-stage probability sampling method to obtain a sample of the population across two rural locations in Bonthe District: the riverine and the mainland. Overall, a total of 393 households across 121 villages were surveyed in the riverine and 397 households across 130 villages were sampled on the mainland. Maternal health, child health and sanitation indicators in Bonthe District were compared using Pearson Chi-Squared test with Yates’ Continuity Correction across the two areas.

**Results:**

Women across the two regions self-reported significantly different uptake of family planning services. Children on the mainland had significantly greater rates of health facility based deliveries; being born in the presence of a skilled birth attendant; completed immunisation schedules; and higher rates of being brought to the health centre within 24 h of developing a fever or a suspected acute respiratory infection. Households on the mainland also reported significantly greater use of treated water and unrestricted access to a latrine.

**Conclusions:**

If the government of Sierra Leone is going to deliver on their promise to free health care for pregnant women and their children, and do so in a way that reduces inequalities, greater attention must be paid to the existing service delivery gaps within each District. This is particularly relevant to health policy post-Ebola, as it highlights the need for more contextualised service delivery to ensure equitable access for women and children.

**Electronic supplementary material:**

The online version of this article (doi:10.1186/s12913-016-1496-1) contains supplementary material, which is available to authorized users.

## Background

Consistently ranked among the countries with the highest maternal mortality ratio (890 deaths per 100,000 live births) [1], Sierra Leone remains one of the worst places on earth for women to give birth [2, 3]. It is estimated that one in every 23 women risk dying during pregnancy and childbirth [4, 5], with many more at risk of complications and lifelong health consequences. In addition to poor maternal health indicators, child morbidity and mortality indicators are also cause for concern with an estimated child, infant and neonatal mortality of 182, 117 and 50 per 1000 live births, respectively [1, 6]. The causes of high rates of maternal and child mortality are multiple and complex, and likely compounded by the fact that the country is still emerging from the effects of a decade-long civil conflict and the devastating effects of the most recent Ebola oubreak.

Helmed by strong political will, the Ministry of Health and Sanitation (MOHS) introduced the Free Health Care Initiative (FHCI) in April 2010. As part of the FHCI, 1.5 million children and their pregnant or lactating mothers were entitled to receive free essential health services, including antenatal, delivery, and postnatal care for mothers, as well as preventative and curative interventions for common childhood illness. Hailed by many at the time as an important step towards improving maternal and child health [7], early reports were quick to highlight the immediate impact of this initiative. Health centres saw a growing number of women and their children accessing health services; women needing urgent care during delivery began attending health centres; and the use of facilities for paediatric surgical cases and common childhood infections including malaria, diarrhoea, and pneumonia reportedly rose by more than 200 % [8–10].

Despite the increase in women and their children accessing health services, there is still good reason to question the sustainability of the FHCI. In 2012, only three obstetricians were registered with the MOHS [3]. Overburdened health staff struggle to keep up with an increasing demand for services and essential medicines [10–12]. With drugs often in short supply, health staff often result to sending patients to buy medicines elsewhere, or charging patients for drugs as a means of cost-recovery [13]. Such out-of-pocket expenditures for women and children go against the very essence of the FHCI and calls to attention that though Sierra Leone has managed to increase the absolute number of women and their children accessing health centres, this does not necessarily translate into reducing inequities in service provision. This is consistent with other progress seen in reducing under-five mortality, where improvements are distributed differently across wealth quintiles [14].

The inequitable distribution of health services and health facilities, a severe lack of health equipment and essential medicines, as well as a shortage of skilled and motivated health staff remain key factors underlying the poor performance of health systems to deliver effective maternal and child health care services in Sierra Leone [13, 15, 16]. There is a need to better understand how the FHCI policy impacts on health inequities in Sierra Leone, and more specifically how this translates into practice and interventions at the community-level. The purpose of this study was therefore to identify current gaps in service delivery across two rural locations, both of which are served by the same District Health Management Team (DHMT). The results of this study were used to inform local policy and adapt maternal and child health programming to better address inequities in health services delivery in Bonthe District.

### Bonthe District, Sierra Leone

Located on the southern-most coast of Sierra Leone, Bonthe District is comprised of 11 chiefdoms and one municipality. One of 13 medical districts in Sierra Leone, Bonthe District serves a population of approximately 160,500 people [17] and is divided into two main geographical regions: the mainland and the riverine. The mainland is comprised of seven chiefdoms including: Bum, Sogbeni, Jong, Imperi, Kpanda Kemoh, Yawbeko, and Nongoba Bullom. The remaining four chiefdoms of Bendu-Cha, Sittia, Dema, and Kwamebai-Krim along with Bonthe Municipality make up the riverine. Both the riverine and mainland are marked by topographical, economical and administerial differences.

While the entire district contains many rivers, estuaries, and lagoons, the riverine is particularly difficult to access by road. With the exception of the roads servicing the bauxite and rutile mines in the chiefdoms of Imperi and Kpanda Kemoh, road conditions are difficult throughout the district and access to most roads is restricted, if not impossible, during Sierra Leone’s rainy season lasting from June to November. Moreover, three of the chiefdoms (Sittia, Dema, and Kwamebai Krim) and Bonthe Municipality, all in the riverine, are only accessible by boat. The topography of Bonthe District predisposes many of its inhabitants to waterborne illnesses including cholera and other diarrhoeal diseases, typhoid, dysentery, schistosomiasis and trachoma, malaria and river blindness. The terrain and remoteness of Bonthe District make it particularly difficult to recruit and retain health workers and as a result, the district is severely understaffed. According to a 2011 Bonthe District DHMT report [17], only 46.3 % of health centres in the district contained one trained and qualified staff, with health centres in the riverine particularly lacking qualified staff.

Economically, approximately 80 % of the District’s total population lives below the poverty line, 90 % of which live in the riverine. Administratively, two local government councils govern Bonthe District: Bonthe Municipal Council, located in the riverine and the Bonthe District Council, located on the mainland in Mattru Jong. While the Government of Sierra Leone (GoSL) provides health resources to the Bonthe DHMT through both the Bonthe Municipal Council and Bonthe District Council, resources are allocated on the basis of population, without consideration for the aforementioned topographical and economical differences. In addition to funding from the GoSL, health centres across both regions receive considerable external support from a number of international and non-governmental organizations.

## Methods

This paper examines where the key gaps exist in the delivery of essential maternal and child health services in Bonthe District. The secondary data analysed for this paper were collected as part of a baseline evaluation for a maternal and child health programme being implemented in Bonthe District as part of the World Vision Sierra Leone, World Vision Ireland, and World Vision UK’s PPA/AIM-Health programme during October-November 2011. As part of AIM-Health/PPA, CHWs are selected, trained and deployed to regularly promote 7 key health intervention messages targeting pregnant women and 11 intervention messages targeting mothers of children under-2. Named the 7–11 strategy, these health intervention messages are delivered through a minimum of ten household visits by a community health worker (CHW).

### Sampling

World Vision’s PPA/AIM-Health programme services two locations and one municipality in the riverine (Sittia, Dema, and Bonthe Municipality) and four chiefdoms on the mainland (Imperi, Jong, Sogbeni, and Kpanda Kemoh). A two-stage probability sampling method was applied to obtain a sample of the population across in the riverine and on the mainland. In the first stage of sampling, a list of all the 199 villages from selected chiefdoms of the mainland and 121 villages of the selected riverine chiefdoms was compiled. The probability of a village being selected was therefore set as proportional to the number of households within that village. In this first stage, all 12,037 households on the mainland and 4712 households in the riverine therefore had an equal chance of being selected regardless of whether they contained the target population or not. The total number of households to include was then calculated assuming a confidence level of 95 % (α = 0.05), with an additional 5 % added to account for non-responses. This resulted in a minimum target of 373 households in the riverine, and 391 households on the mainland.

In the second stage of sampling, village leaders led field teams to the village centre where a pen was spun to determine the field team’s walking direction. A random number generation table was subsequently used to decide which household was to be visited first. A household was defined in terms of persons who were co-resident and shared common cooking arrangements, and were able to recognise one person as the head of household [18]. In the event that residents were absent from their home or that the target group was not present, field teams were instructed to proceed to the “next” household, which was defined as the one whose front door is closest to the one just visited. Enumerators proceeded to the next household, until the total number of households to be sampled from that village was completed.

### Survey tool

The survey tool [see Additional file 1] was developed in consultation with the Bonthe DHMT and with assistance from maternal and child health experts within the World Vision Partnership. A total of 30 community health workers (CHWs) were selected as enumerators to participate in the household survey training, hosted by staff from neighbouring health centres and the DHMT. As part of the survey training enumerators were taught how to administer the questionnaire, record responses from participants, verify patient health cards, interpret the mid-upper arm circumference (MUAC) tapes, and weigh and measure children.

The survey included questions about household demographics; food intake; child health, including symptoms of acute respiratory infection (ARI), diarrhoea, and fevers, as well as treatment at a health centre within 24 h of the aforementioned symptoms’ onset; child vaccination (calculated for appropriate ages including measles at 9 months, OPV at birth, and 9-months for full immunisation, etc.); maternal care services, including Intermittent Preventive Treatment (IPT), use of insecticide treated nets (ITNs), access to antenatal (ANC), postnatal (PNC) and HIV services; delivery in health centres and in the presence of a skilled birth attendant (SBA); use of any family planning method (calculated by household use rather than individual); hand washing and latrine use. To minimise the risk of response bias, answers were verified through child and maternal health cards (i.e., child vaccinations, ANC visits), where available. The presence of a mosquito net and latrine was verified by the enumerator, as was the cleanliness of the latrine. Piloting of the questionnaire took place in villages not included as part of the final sampling frame. Where appropriate, questions were phrased in binomial (i.e., yes or no) format to facilitate the collection of data. Though the questionnaire was printed in English, training was conducted in a mixture of Krio and Mende and CHWs were instructed to conduct the interview in whichever language they felt best suited the household.

### Exclusion and inclusion criteria

To be considered for secondary analysis a household had to contain at least one pregnant woman and/or a child under the age of 5. Interviews were conducted with the child’s primary caregiver, defined as the person who was, “primarily responsible for the health, safety and comfort of that child”. A sample of 393 households across all 121 villages in the riverine, and 397 households across 130 of the 199 villages on the mainland were ultimately represented in the study sample, for a total of 790 households.

### Data analysis

Quantitative analysis was conducted using SPSS Statistics 17 & 22 (Release Versions 17.0.0 and 22.0.0). Using point prevalence data for various binomial variables (and variables which could be transformed into binomial variables), prevalence data were compared across households in the riverine and the mainland using Pearson Chi-Squared test with Yates’ Continuity Correction. To maximise all data available, missing data was handled using pairwise deletion. Rank variables were compared across the two areas using the Mann-Whitney test. Maternal health, child health and sanitation indicators in Bonthe District were compared across two geographically different areas; the riverine and the mainland. All tests were conducted for 95 % confidence with α = 0.05.

## Results

### Response rates

According to the enumerators’ logs, 504 households were visited on the mainland, with 397 surveys collected, indicating a 78.8 % response rate. In the riverine, 491 households were visited and 393 surveys were collected, amounting to a response rate of 80.0 %. Non-responders include those that did not meet the criteria for inclusion as well as those that refused to participate.

### Characteristics of the sample

A total of 205 pregnant women (*n* = 69 in the riverine, *n* = 136 on the mainland) and 1187 children under 5 (*n* = 486 in the riverine, *n* = 701 on the mainland) were represented across the 790 households surveyed. Pearson’s Chi-squared test of significance found no significant differences in age category (*p* = .791) or education level (*p* = .218) for pregnant women across the two locations. Whereas no significant difference was found for age category (*p* = .141), the gender distribution was found to differ significantly (*p* < 0.05) across the two regions for children under-5. During post-hoc analysis however, gender was not found to be a confounding factor across the specific child variables measured and therefore the groups were still considered comparable. Table 1 displays a comparison of the study’s sample characteristics.

**Table 1:** Chi-square values applied to the characteristics of household compared across regions: riverine and mainland, October-November 2011

Respondent characteristic	Riverine	Mainland	Chi-squared	*p*-value
Sex of Head of Household	*n* = 389	*n* = 397		
Male	93.0 %	91.9 %	0.355	.551
Female	6.9 %	8.0 %		
Age of Head of Household	*n* = 359	*n* = 377		
15–18	0 %	0.5 %	1.913	.384
19–49	83.6 %	83.3 %		
50+	16.4 %	16.2 %		
Education Level of Head of Household	*n* = 379	*n* = 383		
None	74.1 %	59.0 %	23.977	.001
Primary	7.4 %	12.3 %		
Secondary or Higher	18.5 %	28.7 %		
Pregnant	*n* = 582	*n* = 744		
Yes	11.9 %	18.3 %	10.31	.001
No	88.1 %	81.7 %		
Age of Pregnant Women	*n* = 69	*n* = 136		
15–18	13.0 %	11.0 %	1.042	.791
19–33	65.2 %	72 %		
34–44	18.8 %	14.7 %		
45+	2.8 %	2.2 %		
Education of Pregnant Women	*n* = 69	*n* = 136		
None	75.4 %	59.6 %	5.754	.218
Some Primary	11.6 %	17.6 %		
Completed Primary	2.9 %	6.6 %		
Some Secondary	10.1 %	14.7 %		
Completed Secondary	0 %	1.5 %		
Sex of Children Under-5	*n* = 486	*n* = 701		
Male	54.5 %	47.6 %	5.209	.022
Female	45.5 %	52.3 %		
Age of Children Under-5 (in years)	*n* = 486	*n* = 701		
<1	18.3 %	21.7 %	6.904	.141
1	22.0 %	17.4 %		
2	22.2 %	19.5 %		
3	18.3 %	20.5 %		
4	19.1 %	20.8 %		

### Maternal health

Of the pregnant women interviewed, 85.3 % of riverine respondents and 84.1 % of mainland respondents had attended at least one ANC visit during their current pregnancy, with no significant differences between the two settings (*p* = .824). Almost half (44.8 %) of women on the mainland reported using a method of family planning compared to less than one-third, (28.4 %) of women in the riverine, with a statistically significant difference between the two locations ([9.8, 23.2] *p* < 0.05).

No differences were found in the number of households who reported a maternal death between the mainland and the riverine, nor were significant differences found in the prevalence of women using folic acid and IPT/ISP. Moreover, there were no significant differences between the number of women who had reportedly slept under an ITN the night preceding the survey; women who correctly identified two methods of prevention of mother to child transmission of HIV (PMTCT); and the number of women who had been tested for HIV during their pregnancy. A summary of the maternal health indicators compared across the mainland and the riverine can be found in Table 2.

**Table 2:** Comparison of maternal health factors across regions: riverine and mainland, October-November 2011

Variable	Riverine		Mainland		Chi-squared	*p*-value
	n	%	n	%		
Maternal Deaths	9	2.3 %	3	0.8 %	2.193	.139
Attended Appropriate Number of ANC	39	57.4 %	76	61.8 %	0.198	.656
Attended all 4 ANC visits	4	5.9 %	24	18.2 %	4.664	.031
Ferrous/Folic	55	84.6 %	109	84.5 %	0.000	1.000
SBA Pre-FHCI Post-FHCI	235 161 74	48.4 % 50.5 % 44.3 %	395 236 159	56.3 % 51.6 % 64.9 %	6.907 0.062 16.302	.009 .804 .000
TBA Pre-FHCI Post-FHCI	247 154 93	50.8 % 48.3 % 55.7 %	312 229 83	44.4 % 50.1 % 33.9 %	4.438 0.185 18.426	.035 .667 .000
Health Centre Delivery Pre-FHCI Post-FHCI	212 143 69	43.6 % 44.8 % 41.3 %	368 218 150	52.4 % 47.7 % 61.2 %	8.552 0.514 15.016	.003 .473 .000
IPT/IPS	50	82.0 %	102	79.7 %	0.030	.862
Dewormed	48	81.4 %	93	74.4 %	0.729	.393
Access to PMTCT	42	63.6 %	78	59.5 %	0.161	.688
HIV Tested	41	62.1 %	90	68.7 %	0.583	.445
Slept Under an ITN	66	95.7 %	131	97.0 %	0.012	.914
ITN was intact	43	65.2 %	86	66.2 %	0.000	1.000
Family Planning	110	28.4 %	173	44.8 %	21.920	.000

#### Place and method of delivery

More than half (52.4 %) of children under-5 on the mainland were reported to have been delivered in a health facility, compared to 43.6 % of children under-5 from the riverine, with children on the mainland having significantly greater health facility based deliveries (*p* < 0.05). Likewise, 48.4 % of children under-5 were delivered by a skilled birth attendant in the riverine, compared to 56.3 % in the mainland. This represents significantly fewer deliveries attended by a skilled birth attendant in the riverine (*p* < 0.05). Children under-5 in the riverine had significantly higher rates of delivery by a Traditional Birth Attendant (TBA) (50.8 %), compared to children on the mainland (44.4 %, *p* < 0.05). Differences in health centre deliveries, and delivery in the presence of both a SBA and TBA were only observed for children born after the introduction of the FHCI (*p* < 0.05, in all cases), see Table 4. While no changes were observed in the riverine, delivery in the presence of a SBA and within a health centre appears to have increased, and the use of TBAs decreased, on the mainland among deliveries occurring after the introduction of the FHCI. There was no difference between the number of children in possession of a birth certificate across the two areas.

### Children under-5

#### Child immunisation

A summary of child health indicators compared across the mainland and the riverine can be found in Table 3. We found no differences in coverage of deworming among children aged 12–59 months or vitamin A supplementation among children aged 6–59 months across the two areas. However, children aged 9–59 months in the riverine had a significantly lower rate of immunisation when compared to children of the same age on the mainland (*p* < 0.05). Only 84.9 % of children in the riverine had been fully immunised, compared to 91.5 % on the mainland. When disaggregated by type of immunisation, significant differences were found across the vaccine schedule, including a difference of 4.1 % (*p* < 0.05) for OPV coverage, 7.1 % (*p* < 0.05) for DPT coverage and 4.8 % (*p* < 0.05) for BCG coverage, with the mainland reporting overall higher vaccination coverage. Only measles had no significant difference in vaccination coverage across the two areas. No differences in immunisation rates were observed for children born pre- or post-FHCI, see Table 4.

**Table 3:** Comparison of child health factors across regions: riverine and mainland, October-November 2011

Variable	Riverine		Mainland		Chi-squared	*p*-value
	n	%	n	%		
Child Deaths	2	0.4 %	19	2.7 %	7.545	.006
Birth Certificate	163	33.5 %	231	33.0 %	0.013	.909
HIV Tested	291	60.4 %	550	80.2 %	54.086	.000
Child (12-59months) Dewormed						
Last 12 months Last 6 months	425 418	92.6 % 91.9 %	548 534	91.5 % 91.9 %	.294 .000	.588 1.000
Child (6–59months) Vitamin A Supplement Last 6 months Last 12 months	428 426 432	92.6 % 93.0 % 93.3 %	573 563 578	94.1 % 94.1 % 94.9 %	0.680 0.387 0.967	.410 .534 .326
Fully immunised Pre-FHCI Post-FHCI	393 273 120	84.9 % 86.4 % 81.6 %	560 413 147	91.5 % 92.0 % 90.2 %	10.840 5.669 4.042	.001 .017 .044
Full OPV	423	90.2 %	612	94.3 %	6.098	.014
Full DPT	414	87.5 %	613	94.6 %	16.893	.000
Full PCV	431	91.5 %	613	94.9 %	4.568	.033
BCG	444	91.7 %	674	96.6 %	12.073	.001
Measles	417	91.4 %	568	93.7 %	1.690	.194
All 3 PNC	299	61.8 %	415	60.1 %	0.284	.594
Slept under net	470	96.5 %	660	96.4 %	0.000	1.000
Net was intact	244	52.5 %	430	65.4 %	18.577	.000
Breastfed	29	78.4 %	61	82.4 %	0.066	.797
MUAC	7	1.5 %	19	3.0 %	1.857	.173
Diarrhoea	24	5.0 %	25	3.7 %	0.903	.342
ARI	32	6.6 %	79	11.6 %	7.569	.006
Fever	53	11.0 %	113	16.6 %	6.952	.008
Treated in last 24 h	36	50.7 %	96	76.2 %	12.214	.000

**Table 4:** Comparison of child and maternal health factors between births before and after the implementation of FHCI within each region; riverine and mainland

Variable	Pre-FHCI		Post-FHCI		Chi-squared	*p*-value
	n	%	n	%		
Fully immunised Riverine Mainland	686 273 413	89.7 % 86.4 % 92.0 %	267 120 147	86.1 % 81.6 % 90.2 %	2.413 1.420 0.293	.120 .233 .588
SBA Riverine Mainland	235 161 236	48.4 % 50.5 % 51.6 %	395 74 159	56.3 % 44.3 % 64.9 %	2.930 1.427 10.859	.087 .232 .001
TBA Riverine Mainland	247 154 229	50.8 % 48.3 % 50.1 %	312 93 83	44.4 % 55.7 % 33.9 %	4.496 2.122 16.368	.034 .145 .000
Health Centre Delivery Riverine Mainland	212 143 218	43.6 % 44.8 % 47.7 %	368 69 150	52.4 % 41.3 % 61.2 %	4.479 .416 11.157	.034 .519 .001

#### Child health

There were no significant differences between levels of reported exclusive breastfeeding for children under 6 months between the riverine and the mainland. For all anthropomorphic indicators including MUAC, weight for age, height for age, and weight for height, no significant differences were found between the two contexts, see Table 3.

Reported levels of fever and presumed ARI among children under-5, as determined by the caregiver’s recall within the last two weeks, were significantly higher on the mainland: 16.6 % of children reportedly experienced a fever, compared to 11.0 % in the riverine, and 11.6 % of children on the mainland, compared to 6.6 % of children in the riverine, experienced symptoms of an ARI. There was a statistically significant difference of 25.5 % (*p* < 0.05) between children who were brought for treatment in the last 24 h between the two areas. In the riverine, 50.7 % of ill children were reported as brought to a health centre within 24 h, compared to 76.2 % of ill children on the mainland.

### Sanitation

There were no differences in the prevalence of households with access to adequate and accessible water, defined as more than 15 L per person per day, with a collection time of less than 30 min. However, there was a significant difference (23.9 %, *p* < 0.05) in households with access to safe and protected water. Nearly a third (31.8 %) of households in the riverine, compared to more than half (55.7 %) of households on the mainland, were using treated water or water from a protected source stored in a clean container. Significant differences (20.0 %, *p* < 0.05) were also seen in the amount of households with unrestricted access to a clean latrine, whereby 21.1 % and 41.2 % of household owned a latrine on the riverine and mainland, respectively. Of those households with access to a latrine, 74.2 % of these were identified as clean on the riverine, a significantly lesser proportion than the 89.6 % identified as clean on the mainland (15.4 %, *p* < 0.05). Table 5 displays a comparison summary of sanitation factors across both regions.

**Table 5:** Comparison of household sanitation factors across regions: riverine and mainland, October-November 2011

Respondent characteristic	Riverine		Mainland		Chi-squared	*p*-value
	n	%	n	%		
Litres (15+)	205	52.3 %	216	57.3 %	1.742	.187
Time (<30)	133	34.2 %	123	31.1 %	0.697	.404
Accessible (15+ & <30)	47	12.1 %	52	13.5 %	0.222	.637
Protected	141	35.9 %	229	57.7 %	36.840	.000
Safe	230	58.5 %	310	78.1 %	34.038	.000
Treated	152	38.7 %	220	56.8 %	25.084	.000
Prot & safe/treat	125	31.8 %	221	55.7 %	44.716	.000
Unrestricted	23	5.9 %	31	8.0 %	1.053	.305
2+ hand wash	357	90.8 %	361	91.2 %	0.001	.973
Latrine	83	21.1 %	163	41.2 %	35.996	.000
Clean latrine	69	74.2 %	155	89.6 %	9.664	.002
Hand wash latrine	43	46.2 %	90	52.0 %	19.005	.000
Kitchen	261	66.4 %	266	67.0 %	0.010	.920
Drying rack	126	32.1 %	148	37.8 %	2.149	.143
Rubbish pit	129	32.8 %	203	51.1 %	16.427	.000

## Discussion

Differences in health indicators across distinct geographic areas have been noted in other contexts [19] and recent studies have called for additional research exploring regional variability in the delivery of maternal and child health services in sub-Saharan Africa [20]. In line with this, our study identifies discrepancies in maternal and child health indicators across two separate contexts, the mainland and the riverine, both served by the same district health management team. Results suggest no significant differences in the demographic make-up of both groups, allowing us to compare them for the purpose of the study. Specifically, we found no differences in age, education and marital status, all of which are well recognised as important determinants of women accessing maternal health services [21–24]. There were however, four main differences across maternal and child health indicators between the riverine and mainland found in this study.

In terms of reproductive health services, women on the mainland reported a higher use of family planning (traditional or modern), compared to women in the riverine. There were also key differences in place of delivery between the riverine and the mainland, with the mainland reporting more frequent facility based deliveries and a greater use of skilled birth attendants. More importantly, further stratification suggests that the differences for children born in a health centre and in the presence of a SBA or TBA only existed for children born after the introduction of the FHCI. In particular, the increased rates were significant on the mainland for health centre and SBA deliveries, and there was a significant decrease in reported TBA presence at births. However, in the riverine there was no difference seen between pre- and post-FCHI. Thirdly, child immunisation rates, with the exception of measles, were higher on the mainland. While differences existed whether children had been born prior to the initiation of the FHCI, the differences were less pronounced for children born after the introduction of the FHCI. Lastly, fewer households in the riverine had access to safe and/or treated water in comparison to households on the mainland. Taken together, our results point to the need for more resources to be put into reproductive, maternal, and child health services in the riverine. Specifically, the Bonthe DHMT should consider directing more resources towards increasing access to family planning, delivery services, and skilled birth attendants. More immunisation efforts are also needed in the riverine and additional investment is necessary to improve water and sanitation services. The following sections draw from the extant literature to explore the potential reasons for these observed differences and make suggestions for how these could be addressed.

A recent systematic review of drivers and deterrents of accessing a health centre for reproductive and maternal health in sub-Saharan Africa identified maternal education, parity, household socioeconomic status, rural or urban dwelling, distance to a health facility and number of ANC visits as the factors most consistently associated with having a facility based delivery [20]. Place of delivery can also be influenced by a women’s social circle [20] and by factors such as the influence and education levels of the head of household [25]. Similarly, attitudinal resistance, awareness of services, societal and cultural pressures, socioeconomic barriers, availability of transport, access to appropriate services, and perceived quality of care are commonly cited barriers to reproductive health services [26–33]. The last three are characteristic of health system failures and of particular relevance to a district health management team.

### Availability of transport and access to appropriate health services

Among other environmental factors, accessibility and availability of transport are commonly noted throughout the literature to impact on health service [34]. In this study, the difficult terrain characteristic of the riverine may partly explain why women in the riverine are more likely to give birth at home rather than in a health centre. The topography of the riverine requires that one navigate a complex system of rivers, lagoons and estuaries both on foot and by boat. With previous studies linking long travel times to increases in child mortality [35–37], it is important that pregnant women access a health centre at the time of delivery. As a recommendation, the DHMT might consider the use of maternity waiting homes, or a facility within reach of a health centre that provides emergency obstetric care [38]. Maternal waiting homes have been employed in other low-income contexts, including neighbouring Liberia, where they were found to be an effective strategy for increasing the use of skilled birth attendants and improving maternal and neonatal health [39]. As, if not more important, than ensuring a safe and clean environment for delivery however, is the availability of a skilled health worker.

In addition to restricting access to care, difficult geographical conditions can also result in poor living and working conditions for health staff. Studies have highlighted the lowered performance of health workers on remote islands such as those represented in the riverine [40] and other remote peripheral health units in Sierra Leone have also shown significant inefficiencies [41]. Poor working conditions in turn, are associated with poor performance, increases in attrition rates, and difficulties recruiting health workers, as individuals prefer not to be posted to remote and difficult locations with poor infrastructure and far away from their family [42–44]. The terrain and remoteness of Bonthe District make it an unpopular location for health workers, with the riverine being particularly understaffed. The DHMT may want to consider additional incentives as a means of enticing health workers to the riverine. Salary top-ups and other non-financial incentives such as providing free housing and further education opportunities were found to impact on the willingness of nurses to work in remote areas of Tanzania [45]. In addition, the DHMT may want to select health workers who are more intrinsically motivated [46] and who originate from very remote areas, as they have been found to express a greater willingness to take up a remote position [45]. To maintain the equitable delivery of MCH services, it is imperative that less resourced areas have in place strategies to maintain a satisfied health workforce.

Widely recognised as effective preventative practices, child immunisation and appropriate water and sanitation are important determinants of maternal and child health [47, 48]. In contexts with scare human resources for health, enlisting the help of alternative cadres of health worker, such as the community health workers (CHWs), is recommended for the effective delivery of preventative maternal and child health care interventions [49]. CHWs are considerably less expensive to train, are selected by the very communities they serve, and with adequate training, can help address many of the cultural barriers often associated with delayed or inconsistent access to health care services [50, 51]. The use of CHWs may be particularly appropriate for the riverine, given its remoteness and difficult topography.

Affordability is also an important factor when considering the equitable access to transport and health services. Though the FHCI theoretically removed user fees, studies report that women have continued to pay for services since the initiation of the scheme [52]. Unfortunately, these findings are not uncommon in free health care initiatives [53, 54], with patients continuing to pay out-of-pocket for transport, medical supplies, and for informal payments requested by health centre staff. These expenses are likely higher in areas such as the riverine, where there are longer distances to travel to the health facility, resources are scarcer or more difficult to obtain, and where the working conditions for health staff are considerably worse. A more recent study conducted in Sierra Leone on the impact of removal of user fees showed that more educated women were more likely to benefit from their removal, with no impact on service utilisation across wealth quartiles [55]. As McKinnon et al.’s [55] review notes, the removal of user fees alone is not enough to reduce inequalities in the delivery of health care. Barriers that impact on socioeconomic inequalities, and access to quality service need to be addressed and included within national plans if Sierra Leone is to implement their FHCI equitably and efficiently amongst its population.

### Perceived quality of care

It is worth noting that along with the introduction of the FHCI, Sierra Leone also made illegal the practice of deliveries being assisted by a traditional birth attendant (TBA) [56]. The decision to give birth in the presence of a TBA is largely influenced by cultural and societal norms, with individuals reporting a more personal relationship, better quality care, and greater trust in TBAs. Similarly, a lack of available or inaccessible health facilities and/or a poor opinion of hospital services and staff also contribute to a preferred use of TBA [31, 57]. TBAs are also considered to be more affordable, as they are amenable to being paid in-kind or by instalments [58]. These factors may all contribute to our observation of greater TBA use and a greater prevalence of women giving birth at home in the riverine.

While the Bonthe DHMT is well aware of the legal standing of TBAs, they must acknowledge the value that some women place on TBAs. In the absence of a greater number of trained health providers posted to the riverine, TBAs remain an important resource for the transmission of health messaging and to increase service utilisation. For example, TBAs are an important resource to encourage pregnant women to come to the health centre to attend antenatal services and when engaged appropriately, and like community health workers [49], can act as an important bridge between communities and more formal health systems [39]. Consideration should be given for how to best engage TBAs in Bonthe District, as an important strategy to achieve a more equitable maternal health care service.

### Equity, not equality

While the Government of Sierra Leone (GoSL) currently provides health resources to the Bonthe DHMT through both the Bonthe Municipal Council (located in the riverine), and Bonthe District Council (located on the mainland), resources are allocated on the basis of population with little consideration for typography, difficulty of access, and the demographic profiles of potential health care users. The results of this study indicate that the riverine may be disproportionately disadvantages by a lack of resources. Contingencies for areas of greater need should be developed and implemented, which includes increased availability of skilled health workers, more focused immunisation campaigns, and greater resources to address the lack of access to water and sanitation. In other words, initiating a free MCH programme alone is not enough to reduce disparities within populations and resources in Bonthe District should be allocated to specific health centres on the basis of need, rather than on the basis population. In addition, routine monitoring of national and district data should be conducted to capture “health systems input, quantities and prices and health services outputs to facilitate regular efficiency analyses” [41]. Early involvement of stakeholders is particularly important in the early stages of MCH programmes as they can provide important suggestions for programme re-alignment, and how to meet end-user expectations [59]. Helping programmes better plan for service delivery is important to ensure access to care for more vulnerable populations and to deliver equitable health services. Additional research into the reasons for the observed discrepancies will be important to further inform the implementation of health activities and the distribution of resources going forward.

### Sierra Leone post-Ebola

Data from this study was collected prior to the Ebola outbreak that afflicted Sierra Leone and its neighbouring countries of Guinea and Liberia. The recent outbreak resulted in over 3950 deaths in Sierra Leone alone [60] and has left an already under-resourced and overstretched health system in a severe crisis. With 6.85 % of Sierra Leone’s health workforce being lost to the Ebola virus, some predict an increase in maternal and infant mortality of 74 and 13 %, respectively [61]. The tragic loss of health staff has the potential to result in an estimated 4022 additional women dying during pregnancy and childbirth per year across Sierra Leone, Guinea and Liberia, reminiscent of levels during the civil conflict [62].

Findings during the outbreak have indicated a decrease in RMCH service utilization [63], with evidence that Ebola has disproportionately affected women and children [64]. In the aftermath of Ebola, it is predicted that its devastating consequences will again most be felt by women and children [61]. It is therefore not unreasonable to assume that the discrepancies observed in this study will be exacerbated as a result of the recent Ebola outbreak, causing a further decay of the health care system and an even greater shortage of health workers.

The challenge for Sierra Leone will be to rebuild and strengthen its health system in an efficient and equitable manner. As these findings show, considering existing discrepancies and contextual conditions, over and above populations numbers, are essential to ensure equitable – not just equal - distribution of maternal and child health resources.

### Limitations

This study is not without its share of limitations. Notably, sampling errors may have occurred during the development of the sampling frame, impacted by the non-availability of community maps. Similarly, only households where someone was present during the day were sampled, excluding houses where people were away working or caring for their farms. In addition, a pocketing effect may have been introduced due to the random number generators for first house selection. This pocketing effect could influence certain indicators, such as immunisation rates. Bias may also have been introduced during the data collection process. This includes social-desirability bias, which has been noted in other studies regarding reporting of breastfeeding and child health indicators and recall bias, for those indicators (fever, diarrhoea, ARIs) that were collected based on the primary caregiver’s perception of illness without validation by a medical professional. In addition, this survey was conducted during the dry season, which may impact on the reported incidence of certain diseases, notably diarrhoea, which is more prevalent during the rainy season.

## Conclusions

As we move towards progressing the Sustainable Development Goals (SDGs), the challenge remains to identify specific geographical inequities in service requirements and establish context specific strategies to increase access to maternal and child health care for less accessible populations, for whom health resources are often offered at a premium. Though Sierra Leone has seen a significant increase in health-care use since 2010, the capacity of the health system to respond to this increased demand for services in the wake of Ebola remains uncertain. As a result, the most vulnerable populations of children and women are not equitably benefiting from national efforts such as the FHCI to improve maternal and child health. This paper provides one example of how two distinct contexts covered by the same health district management team can demonstrate very different indicators for maternal and child health. More context-specific approaches are necessary in Bonthe District in order to address the inequities in maternal and child health indicators highlighted in this study. Similarly, if the government of Sierra Leone is going to deliver on their promise to free health care for pregnant women and children greater attention must be paid to the existing service delivery gaps within each District.

## Abbreviations

ANC, antenatal clinic; DHMT, District Health Management Team; FHCI, Free Health Care Initiative; MCH, maternal and child health; SDG, Sustainable Development Goal; NGO, non - governmental organisation; TBA, traditional birth attendant.

## Additional file

Additional file 1 Household Survey Instrument. (PDF 481 kb)
