# Supplementary material for: Can Sierra Leone maintain the equitable delivery of their Free Health Care Initiative? The case for more contextualised interventions: results of a cross-sectional survey
Source: BMC Health Serv Res. 2016 Jul 13;16:258. doi: 10.1186/s12913-016-1496-1 (PMC4942899; doi:10.1186/s12913-016-1496-1)
Supplement: Additional file 1 — Household Survey Instrument. (PDF 481 kb) [file 12913_2016_1496_MOESM1_ESM.pdf]

HOUSEHOLD REGISTER

Survey Number__________________	Village _______________ Circle: KKS or JONG or IMPERI or SHERBRO	Name of Person completing interview ______________________
Name of CHW member: _____________	Reporting Period (Date) _______________	Head of Household ___________________
RC Present____	Nearest Health Centre:_________________________ Score: **1** (very poor) **2** **3** **4** **5** (excellent)	
Referral (give reason) ________________	Is the respondent aware of any community case management programme active in the area? YES or NO.<br>If yes, which of the following services do they provide (Circle all that apply):<br>MALARIA DIARRHEA ACUTE RESPIRATORY INFECTIONS	Please write the number of women in this household who have died within 40 days of giving birth in the last year: ______
# of People in the Household__________		
Supervisor________________		

Adults & Children Above 5

GENERAL INFO: Adults								PREGNANCY															MALARIA		NUTRITION						DANGER SIGNS								FP	
Q1	Q2		Q3	Q4	Q5	Q6	Q7	Q8	Q9	Q10	Q11				Q12	Q13	Q14	Q15	Q16	Q17	Q18	Q19	Q20	Q21	Q22	Q23					Q24	Q25	Q26	Q27	Q28	Q28b	Q28c	Q28d	Q29	29a
Household Members (HHH & Women of Childbearing Age)	Sex		Age (yrs)	Relationship to HHH	Disability?	Source of Income	Level of Education	Pregnant?	Health Card?	Delivery Month	ANC Visits				TT dose	Ferrous/Folic	IPT/SP	Dewormed?	Access to PMTCT	Received HIV info during ANC	Tested as part of ANC?	Received Results	Slept under ITN last night	Does net have holes?	# Meals Yesterday	Cereals & Starches	Protein	Legumes	Greens	Fruit	# Pregnancy Danger signs	# Neonatal Danger Signs	# Methods of MTCT	# Post Partum Danger signs	# Pneumonia Danger signs	Cause of Polio	Cause Malaria	Cause of Measles	Family Planning?	FP Method
	M	F									1	2	3	4																										

RESPONDENT ONLY

General Information							Pregnancy Care							PNC			Immunisation & Supplements																	Malaria		Nutrition									
Q30	Q31		Q32	Q33	Q34	Q35	Q36	Q37	Q38	Q39	Q40	Q41	Q42	Q43	Q44	Q45	Q46	Q47	47b	47c	47d	47e	47f	47g	47h	47i	47j	47k	47l	47m	Q48		Q49		Q50	Q51	Q52	Q53	Q54					Q55	Q56	Q57
	Sex																														Worm		Vit A													
Household Members (5 Years and below)	M	F	Age (months)	Relationship to HHH	Death (past 1 yr)	Disability?	Delivered with SBA?	Delivered with TBA?	Delivered in HC	Birth Certificate?	Received HIV info during ANC	Tested as part of ANC?	Received Results	6 hours	5-7 days	6 weeks	Has Card?	BCG	OPV 0	OPV 1	DPT-Heb B+Hib 1	PCV 1	OPV2	DPT-Heb B+Hib 2	PCV 2	OPV 3	DPT-Heb B+Hib 3	PCV 3	Yellow Fever	Measles	1st	2nd	1st	2nd	ITN	Net with holes	Exclusive Breastfeeding	# Meals Yesterday	Cereals & Starches	Protein	Legumes	Greens	Fruit	MUAC (G,Y,R)	Height (cm)	Weight (kg)
																	Birth		6 Weeks			10 Weeks			14 Weeks			9 Months																	

Survey Number___________________

ICCM SICK PATIENT REGISTER FOR CHILDREN 5 YEARS AND BELOW												
GENERAL INFO				PROBLEM (past 2 weeks)					TREATMENT			
Q58	Q59		Q60	Q61	Q62	Q63	Q64	Q65	Q66	Q67	Q68
Patient Name	Sex		Age in months	Diarrhoea	Fast Breathing	Cough	Fever	Treated at HC within 24 hrs	ORS/ORT	Breastfeeding	Home Solution
	M	F									

HOUSEHOLD								
WATER AND SANITATION								
Q69	Q70	Q71	Q72	Q73				
Time Spent Fetching Water (MINS)	# Litres per Day	How is the HH waste disposed?	Water Treatment	*Before Eating*	*After Changing Baby*	*After Latrine*	*Before Food Prep*	*Before Feeding Child*
				5 CRITICAL TIMES				

HOUSEHOLD: Water, Food and Sanitation

Find out if the household has unlimited access to the following methods to prevent sickness. Tick the boxes for the things the family has:

Q74 ☐	Q75 ☐	Q76 ☐	Q77 ☐	Q78	Q79	Q80
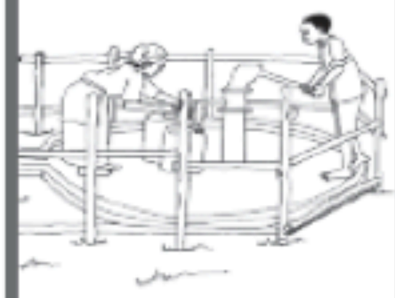	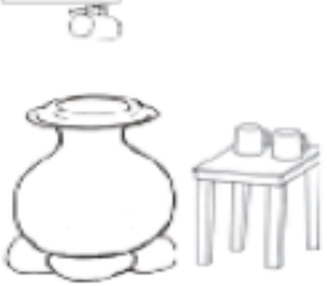	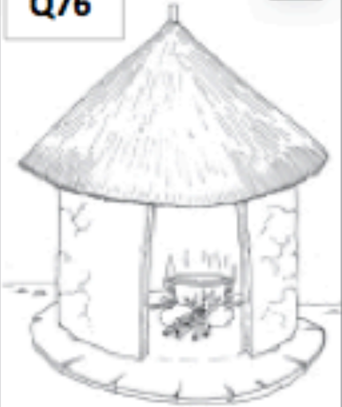	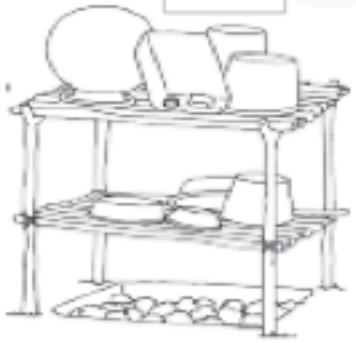	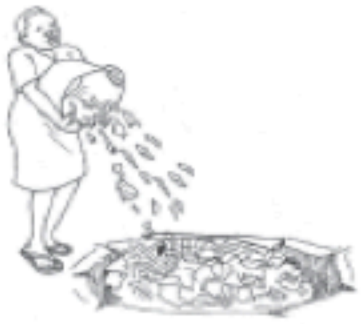	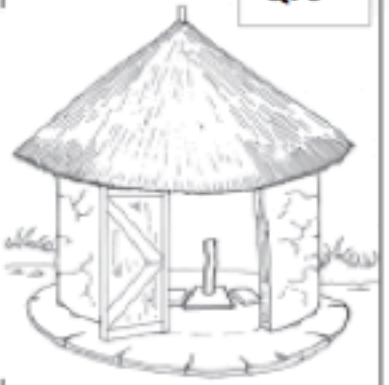	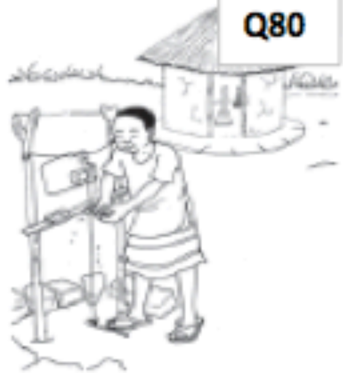
**Protected water source**	**Safe drinking water**	**Kitchen**	**Drying rack for dishes**	**Rubbish pit**	**Latrine**	**Hand-washing area with soap near latrine**

Follow up on anything the family is missing.

LATRINE		
Q81	Q82	Q83
Is Latrine Clean?	Type of Latrine	Where defecate if no latrine?
